# Height is a predictor of hamstring tendon length and ACL graft characteristics in adolescents

**DOI:** 10.1186/s12891-023-06705-2

**Published:** 2023-07-11

**Authors:** Martijn Dietvorst, M. C. Marieke van der Steen, Marijn van den Besselaar, Rob PA Janssen

**Affiliations:** 1 Department of Orthopaedic Surgery and Trauma, Máxima MC, Eindhoven, the Netherlands; 2 Department of Orthopaedic Surgery and Trauma, Catharina Hospital Eindhoven, Eindhoven, the Netherlands; 3 Dept. of Paramedical Sciences, Chair Value-Based Health Care, Fontys University of Applied Sciences, Eindhoven, the Netherlands; 4 Department of Biomedical Engineering, Orthopaedic Biomechanics, Eindhoven University of Technology, Eindhoven, the Netherlands

**Keywords:** Hamstring tendon, ACL reconstruction, graft, anthropometrics, adolescents

## Abstract

**Background:**

Knowing the potential hamstring tendon length is relevant for planning ligament reconstructions in children and adolescents, as it is not uncommon to encounter small hamstring tendons intraoperatively. The aim of this study is to predict semitendinosus and gracilis tendon length based on anthropometric values in children and adolescents. The secondary aim is to analyse hamstring tendon autograft characteristics in a closed socket anterior cruciate ligament reconstructions and to evaluate the relationship with anthropometric variables. The hypothesis of this study was that height is predictor of hamstring tendon length and thereby graft characteristics.

**Methods:**

This observational study included two cohorts of adolescents undergoing ligament reconstructions between 2007–2014 and 2017–2020. Age, sex, height and weight were recorded preoperatively. Semitendinosus and gracilis tendon length and graft characteristics were measured intraoperatively. Regression analysis was performed on tendon length and anthropometric values. Subgroup analyses of the closed socket ACL reconstruction were performed and the relation between anthropometric values and graft characteristics were analysed.

**Results:**

The population consisted of 171 adolescents from 13 to 17 years of age, with a median age of 16 years [IQR 16–17]. The median semitendinosus tendon length was 29 cm [IQR 26–30] and gracilis tendon length was 27 cm [IQR 25–29]. Height was a significant predictor of semitendinosus and gracilis tendon length. Subgroup analysis of the closed socket ACL reconstructions showed that in 75% of the procedure, the semitendinosus tendon alone was sufficient to create a graft with a minimum diameter of 8.0 mm.

**Conclusions:**

Height is a significant predictor of semitendinosus and gracilis tendon length in adolescents between 13 and 17 years of age and outcomes are similar to data in adults. In 75% of the closed socket ACL reconstructions, the semitendinosus tendon alone is sufficient to create an adequate graft with a minimum diameter of 8 mm. Additional use of the gracilis tendon is more often necessary in females and shorter patients.

**Level of evidence:**

3

## Background

Anterior cruciate ligament (ACL) injury in children and adolescents is a severe injury of the knee. The ACL reconstruction technique for children and adolescents depends on skeletal maturity and is also surgeon dependent [1, 2]. However, general principles, such as the use of a well-positioned soft-tissue autograft with an adequate size, diameter and fixation for ACL reconstruction in adults also apply for the younger patient population [1, 3]. Desired graft length depends on the type of ligament reconstruction, operative technique, for example full tunnels versus closed-sockets techniques, and fixation methods [4]. However, the graft diameter is important regardless of the technique, as a diameter of less than 8 mm is related to higher revision rates within the age category of 20 years and younger [5–11].

Quadrupled hamstring autograft is most commonly used as soft tissue graft for ACL reconstruction in children and adolescents [3]. Both semitendinosus (ST) as semitendinosus-gracilis (STG) grafts are used for ACL reconstructions. In some children and adolescents, the harvested hamstring graft might be too small to produce a graft with adequate specifications [8]. Preoperative knowledge of potential tendon dimensions could assist in graft planning for knee ligament surgery, as complex knee ligament reconstructions require specific tendon length and diameter [12]. Various studies on the anthropometric predictability of tendon dimensions have been conducted in adults, reporting that height is a predictor of hamstring tendon length [12–14]. The question remains whether these outcomes are applicable in adolescents, as adolescence is a period of growth, development and maturation. To this date, one study evaluated the predictability of hamstring tendon dimensions in children and adolescents based on magnetic resonance imaging (MRI) measurements [15]. Other studies evaluated the relationship of anthropometric values or MRI measurements with the diameter of a hamstring tendon autograft for a specific ACL reconstruction technique, but did not analyse the length of the hamstring tendons itself [16–19]. Predictability of hamstring tendon lengths in a paediatric and adolescent population has not been studied to this date, despite that tendon length is also an important aspect to be able to obtain the desired graft dimensions during ACL reconstruction or planning for multiligament reconstructions.

The primary aim of this study is to analyse the preoperative predictability of the ST and G tendon lengths based on anthropometric data in adolescents. We hypothesize that height will be a relevant anthropometric predictor. As a secondary aim this study analyses anthropometric characteristics in relation with graft characteristics, such as ST or STG graft, length and diameter in a closed socket ACL reconstruction technique. We hypothesize that graft characteristics in a closed socket ACL reconstruction, such as additional use of G tendon for graft (ST versus STG graft), can also be predicted by height due to the predictability of tendon length by height.

## Methods

In this retrospective study of prospectively obtained data, consecutive adolescent patients with ACL rupture, scheduled for ACL reconstruction between 2007–2014 and 2017–2020, were eligible for inclusion. In both periods, preoperative height, weight, BMI, sex and age were recorded and hamstring tendon lengths and graft characteristics were described intra-operatively. In the period from 2014 to 2017, the hamstring tendon lengths were not measured and patients from that period were therefore not included in this study. The patients included between 2007 and 2014 were also part of the data analysed in the study by Janssen et al. [12] and in the current study a subgroup analysis on the younger patients (< 18 years) of that cohort was performed [12]. From 2007 to 2014, a full tunnel ACL reconstruction was used. From 2017, a closed socket technique (All-Inside, Arthrex®, Naples, USA) was used for ACL reconstructions. Anthropometric values, hamstring tendon lengths and graft characteristics were recorded again from 2017. All patients (< 18 years) undergoing primary reconstruction with hamstring tendon autografts were eligible for inclusion. Exclusion criteria were ACL reconstruction with other types of allo- or autografts, previous harvest of the ipsilateral hamstring tendon, congenital limb deficiency that would affect total body weight and neuromuscular disorders. Preoperatively sex, age, height and weight of the patient were recorded as anthropometric variables.

Two orthopaedic surgeons (RJ and MvdB) performed all procedures using the same technique. From 2007 to 2014, a STG autograft with WasherLoc™ (Zimmer Biomet®, Warsaw, Indiana, USA) was used as the primary graft for a full-tunnel ACL reconstruction. The methodological description of that period has been published previously [12, 20].

From 2017, a closed socket technique (All-Inside, Arthrex®, Naples, USA) was used to reconstruct the ACL in young ACL reconstruction patients with the ST as autograft type of choice. The ST tendon was harvested and prepared in a standardized fashion according to the previous study [12, 20]. The available length of each tendon was measured with a ruler and recorded in cm, rounded off to the nearest 0.5 cm. A provisional ST graft was then created and the length and diameter of the graft were measured. The diameter of the hamstring graft was measured by soft tissue graft caliper (Arthrex®, Naples, Florida, USA) with 0.5-mm increments between holes and the length of the graft is measured with a ruler. In case of insufficient graft diameter of the ST graft, the G tendon was also harvested. The primary goal was to create a hamstring autograft with a minimum length of 6 to 6.5 cm and diameter of ≥ 8 mm, preferably as a 4-strand ST graft (4-ST). In order to create a 4-ST graft of 6 cm, a minimum ST tendon length of 24 cm was necessary. Depending on length of the tendons and/or diameter of the graft, strand variations of the graft are possible with or without the use of the G tendon. The possible variations were a 3-strand ST, 4-strand ST, 5-strand ST(G), 6-strand STG, 7-strand STG or 8-strand STG graft.

### Statistical analyses

Descriptive analyses were used to describe the groups based on the ACL reconstruction technique. Baseline parameters were compared between groups by medians of the Mann Whitney U test after tests for normality for the continuous variables and Chi square test for sex. Multivariate linear regression analyses were performed to predict the length of harvested ST and G tendons length. As variables of interest the anthropometric parameters sex, age, height, weight and BMI were considered. In order to create a standard 4-ST graft a minimum of 24 cm ST tendon length is necessary, therefore the division between significant anthropometric values and ST tendon length of 24 cm was analysed. Additional analyses were performed on the 2017–2020 closed socket technique subgroup. A logistic regression analysis was performed to analyse the necessity for additional use of the G tendon in the 2017–2020 group. Data analysis was performed in SPSS Statistics version 22.0 (IBM, Armonk, New York, USA). Significance was set at ≤ 0.05 in all analyses.

### Ethical approval

The Institutional Review Board (IRB) of The Medical Ethical Committee Máxima Medisch Centrum determined that this study was not subjected to the guidelines of the Medical Research Involving Human Subjects Acts (WMO) (N20.038).

## Results

**Fig. 1:**
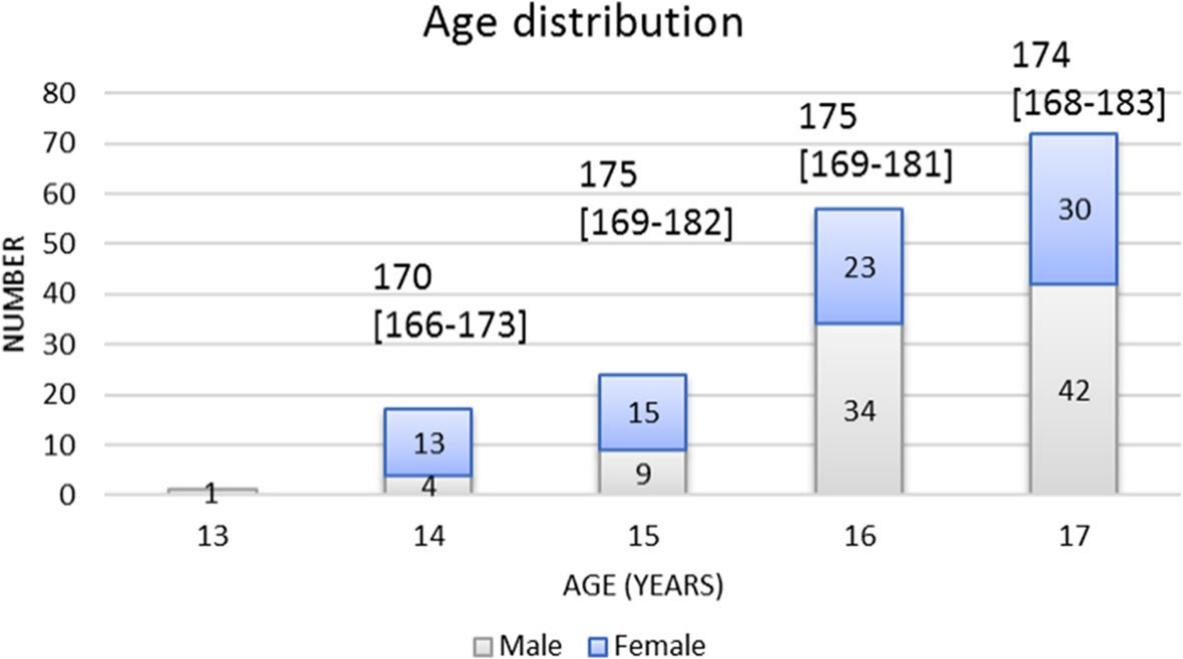
Age distribution of females and males. Numbers above columns represent median height in cm [IQR] within the age-group

A total of 171 patients were included for analysis, of which 99 patients were included in the period from 2007–2014 and 72 patients from 2017–2020. See Table 1 for baseline characteristics and Fig. 1 for the age distribution with median heights. The height, weight and BMI of 2 patients were unknown and of 1 patient the ST length was unknown.

**Table 1:** Characteristics of the study population

	Total	2007–2014	2017–2020	
	*N* = 171 Median [IQR]	*N* = 99 Median [IQR]	*N* = 72 Median [IQR]	*p*-value
Age (years)	16.0 [16.0–17.0]	16.0 [16.0–17.0]	16.0 [15.0–17.0]	0.114
Sex, female %	47%	47%	49%	0.931
Height (cm)	174 [168–182]	174 [168–183]	172 [168–182]	0.354
Weight (kg)	65.0 [60.0–73.5]	65.0 [61.0–74.0]	65.0 [58.0–72.0]	0.388
BMI (kg/m^2^)	21.5 [19.8–23.8]	21.5 [20.1–23.7]	21.4 [19.5–24.0]	0.664
ST length (cm)	29.0 [26.0–30.1]	29.0 [27.0–31.0]	28.5 [26.0–30.0]	0.238
G length (cm)	27.0 [25.0–29.0]	28.0 [25.0–29.5] *N* = 99	24.0 [23.0–27.0] *N* = 18	0.003

### *IQR* interquartile range

#### Prediction of tendon lengths

The multivariate linear regression analyses in the total group on the ST and G tendon lengths are shown in Table 2. Height is a predictor for both the ST and G tendon length. For each centimetre increase in height, the predictive length of the ST and G tendon increase 0.18 cm and 0.14 cm respectively.

**Table 2:** Multivariate linear regression analysis of anthropometric values and ST or G length

Multivariate						
	Semitendinosus tendon length			Gracilis tendon length		
Model	Regression coefficients (95% CI)	*P*-value	R^2^	Regression coefficients (95% CI)	*P*-value	R^2^
Constant	0.702 (-11.848; 13.252)	0.912	0.337	5.223 (-12.456; 22.921)	0.559	0.212
Age (years)	-0.135 (-0.551; 0.281)	0.522		-0.164 (-0.807; 0.408)	0.616	
Sex (female)	-0.876 (-1.978; 0.225)	0.118		-0.619 (-2.132; 0.894)	0.420	
Height (cm)	0.180 (0.115; 0.245)	< 0.001		0.140 (0.053; 0.226)	0.002	
Weight (kg)	-0.016 (-0.059; 0.028)	0.476		0.004 (-0.054; 0.062)	0.898	

*CI* Confidence interval

In order to create a standard 4-ST graft, the required ST tendon length was 24 cm. In Table 3 the division between ST tendon lengths of 24 cm or more and height categories are shown, as height was a significant predictor of ST tendon length.

**Table 3:** Relation of height categories and ST tendon length

	ST tendon length	
Height (cm)	< 24 cm N (%)	≥ 24 cm N (%)
< 160	4 (44)	5 (56)
160–170	5 (13)	33 (87)
170–180	3 (4)	65 (96)
≥ 180	0 (0)	53 (100)

### Closed socket subgroup analysis

In the 2017–2020 group, 75% of the closed socket ACL reconstructions were performed with a ST tendon autograft only. In 18 (25%) cases, an additional G tendon autograft was necessary to achieve adequate graft dimension. Patients with a STG graft were significantly more often female, were shorter and lighter and had shorter ST tendons as is shown in Table 4.

**Table 4:** Characteristics of patient with a ST and STG graft from the 2017–2020 closed socket group

	Patients with ST graft	Patients with STG graft	
	*N* = 54 Median [IQR]	*N* = 18 Median [IQR]	*P*-value
Age (years)	16.0 [15.0–17.0]	16.0 [15.8–17.0]	0.402
Sex, female %	41%	72%	0.021
Height (cm)	175 [169–182]	170 [165–175]	0.032
Weight (kg)	65.0 [60.0–74.0]	58.5 [53.5–67.8]	0.021
BMI (kg/m^2^)	21.8 [19.6–24.1]	20.4 [19.1–23.1]	0.352
ST length (cm)	29.0 [26.0–30.0]	25.8 [24.0–28.1]	0.001
G length (cm)	NA	24.0 [23.0–27.0]	NA
Graft diameter (mm)	8.5 [8.0–9.0]	8.5 [8.0–9.0]	0.568
Strand type % (n)			
3-strand	2% (1)	0% (0)	
4-strand	85% (46)	0% (0)	
5-strand	13% (7)	0% (0)	
6-strand	0% (0)	73% (13)	
8-strand	0% (0)	22% (4)	
10-strand	0% (0)	6% (1)	

*IQR* Interquartile range, *NA* Not applicable

Four of the 72 patients (6%) had a graft diameter of less than 8 mm (all had 7.5 mm), which were all ST grafts and these graft configurations were accepted intra-operatively. Four strand ST graft was used most frequently. In case of using an STG graft, a six strand variation was used most frequently.

The results of the univariate logistic regression analyses of height and additional G tendon use as hamstring autograft is shown in Table 5. The STG group consisted of 18 patients and therefore no stable multivariate model could be created. Due to the significance of height in previous analyses, height was chosen as a factor of interest. According to the univariate logistic regression analysis, patient’s height is a statistically relevant predictor for additional need for the G tendon as autograft.

**Table 5:** Univariate logistic regression analysis of height and additional G tendon use (0 = no additional G tendon use; 1 = additional G tendon use)

Univariate			
Model	Regression coefficient (95% CI)	*P*-value	Nagelkerke R^2^
Constant	11.514	0.056	0.095
Height (cm)	-0.073 (0.868–0.996)	0.038	

*CI* Confidence interval

Patients with an STG graft were significantly more often females. Differences in height and weight could therefore be the result of differences in sex between groups and not to differences in BMI, as BMI was similar in both groups. Males had a significantly greater median height than females (180 cm [IQR 175–185] versus 168 cm [165–170], *p*-value < 0.001). Males also were heavier than females (68 kg [60–79] versus 63 kg [55–67], *p* = 0.009). Univariate logistic regression analysis of height and additional G tendon use within females (*n* = 35, of which *n* = 13 having a STG graft) showed no statistically significant value of height as is shown in Table 6.

**Table 6:** Univariate logistic regression analysis of height and additional G tendon use (0 = no additional G tendon use; 1 = additional G tendon use) within the female subgroup

Univariate			
Model	Regression coefficient (95% CI)	*P*-value	Nagelkerke R^2^
Constant	12.202	0.264	0.056
Height (cm)	-0.076 (0.815–1.053)	0.244	

*CI* Confidence interval

## Discussion

Height is a significant predictor of ST and G tendon length in the age category of 13 to 17 years. This study is the first study to analyse the relationship between anthropometrics, hamstring tendon lengths and graft characteristics in a closed-socket ACL reconstruction. Knowing the potential ST and G tendon length is relevant for planning the hamstring tendon autograft for ligament reconstructions in adolescents [8, 12]. Complex knee ligament reconstructions require specific tendon lengths to create the desired graft dimensions and there are concerns that in some adolescents, the harvested hamstring graft can be too small to produce a graft with an adequate diameter [8, 12]. To this date, no study previously analysed the prediction of hamstring tendon lengths focusing on an adolescent population. The results of this current study are in accordance with the first hypothesis and the study by Janssen et al. [12], which showed that for each increase in 1 cm in body height, the ST and G length increase respectively with 0.20 cm and 0.16 cm in a population with a mean age of 28.7 years [12]. The importance of this study is that hamstring tendon length can be predicted by preoperative body height and hamstring tendons were in all cases large enough to create grafts with a diameter > 8 mm.

Considering that adolescents experience a growth spurt it might be surprising that age was not a significant predictor for tendon lengths in the multivariate regression analyses. However, considering the large variation in onset and duration of this growth spurt [21], maturation of adolescents seems best captured by height instead of age. Furthermore, the great majority of the population were 16 and 17 year-olds and may therefore have reached final height [22]. The increase in height is not only caused by growth of the lower extremities, but also by spinal growth. It is not known whether the increase in height of the lower extremities would result in a relatively equal increase in hamstring tendon lengths. There is limited evidence of the development of human tendons in vivo [23]. Current available evidence shows that throughout childhood and adolescence, the tendons seem to adapt in size and structure as the musculotendinous structures develop [24–27]. The influence of growth on hamstring tendon lengths has not been evaluated previously. However, the influence of growth on Achilles and patellar tendon lengths have been studied and showed that the lengths of both tendons in 14 year-olds boys are similar to adults [28, 29].

Current ACL reconstruction techniques allow the use of multiple-stranded hamstring autografts and depending on the reconstruction technique, different socket/tunnel lengths require different graft lengths [12]. For example, in order to create a 4-strand ST with a minimum length of 60 mm, a minimum ST tendon length of 24 cm (4 × 60 mm) is necessary. In 44% of the patients with a height of < 160 cm the ST tendon was shorter than the required 24 cm. It is necessary in those cases to harvest an additional G tendon, as a 3-ST graft in most cases did not reach the required diameter. A recent systematic review showed that the hamstring tendon graft diameter should be ≥ 7 mm, but a threshold towards larger graft diameters should be considered for patients younger than 20 years [30]. High graft failure rates are problematic in this young population [31]. A graft diameter of less than 8 mm is related to higher revision rates within the age category of 20 years and younger [5–11]. For each increase of 0.5 mm in diameter within the 7.0 to 9.0 mm range, the likelihood of a revision was 0.82 lower [11]. It is therefore important to reduce the risks of graft failure by creating a graft with an adequate length and diameter [9]. Preoperative prediction of tendon length might therefore help in preparing the graft for ligament reconstructions.

In the current cohort of the closed socket technique, 25% of the reconstructed ACL autografts required an additional G tendon to create a graft of sufficient diameter. All STG grafts had a diameter of more than 8 mm and did not need augmentation of contralateral hamstring tendon autograft or allograft. This finding is somehow similar to the outcomes of the study by Stergios et al. [32], who found that the ST tendon alone was insufficient to create a 4-strand graft with a minimum diameter of 7 mm in one in five adult patients [32]. In the current study, logistic regression analysis showed that additional use of the G tendon can be predicted by height, which was in accordance with the second hypothesis. However, as there were significantly more females in the STG group who were significantly smaller than males, subgroup analysis of females showed no significant effect of height. The effect of height on additional use of the G tendon might be explained by the findings that more females needed an additional G tendon and females were smaller than males. This is in line with previous literature showing that females more often had an inadequate ST tendon length to create a ST 4-strand graft and alternative graft options should be considered [18, 32].

This study has several limitations. The first limitation is that the diameter of the tendons is not measured, although most likely both tendon length and diameter contribute to graft size. Recent studies showed that anthropometric data and CSA measurements of hamstring tendon on MRI are correlated to the diameter of hamstring grafts [17–19, 33]. Another limitation of this study is that, similar to the study by Calvo et al. [16], measurements were based on chronological age and not on physiological age [16]. The number of children with remaining growth of the lower extremity is therefore not known. In these cohorts, Tanner stages were not obtained. As the majority of the cohort is 16 and 17 years of age and youngest patients are 13 years of age, it is expected that most patients (especially females) have reached Tanner stage 4 or 5, which means that no or very limited residual skeletal growth is expected. This is in accordance with the median heights in our cohorts, which were not statistically different between different age categories. The influence of physiological age and residual growth on tendon characteristics was not investigated, which would be an interesting topic for future research. Future research should also aim at skeletally immature children specifically of which bone age and remaining growth of the lower extremity is known. Finally, this study population consisted of Caucasian adolescents. Chiang et al. [13] concluded in their study that Caucasian patients had significantly longer hamstring tendons compared to Chinese Han population [12, 13]. The outcomes of this study might therefore not be extrapolated to adolescents of other ethnicities.

## Conclusions

Height is a significant predictor of ST and G tendon length in adolescents between 13 and 17 years of age and outcomes are similar to data in adults. In 75% of the closed socket ACL reconstructions, the ST tendon alone is sufficient to create an adequate graft with a minimum diameter of 8 mm. Additional use of the G tendon is more often necessary in females and shorter patients.

## Data Availability

The datasets used and/or analysed during the current study available from the corresponding author on reasonable request.

## Abbreviations

ACL: Anterior Cruciate Ligament
BMI: Body Mass Index
CM: Centimetre
CSA: Cross-Sectional Area
G: Gracilis
IQR: Interquartile Range
IRB: The Institutional Review Board
KG: Kilograms
M: Meter
MRI: Magnetic Resonance Imaging
N: Number
ST: Semitendinosus
STG: Semitendinosus Gracilis combined
